# Supplementary material for: Prenatal cannabis smoke exposure alters placental development in a murine model of pregnancy
Source: PLoS One. 2026 Mar 16;21(3):e0328123. doi: 10.1371/journal.pone.0328123 (PMC12991273; doi:10.1371/journal.pone.0328123)
Supplement: S1 Table — aCyp1a1, cytochrome P450 family 1 subfamily A polypeptide 1; Pl2, placental lactogen 2; Tfap2c, transcription factor AP-2 gamma; Tpbpa, trophoblast specific protein alpha; Pcdh12, procadherin 12; Igf1r, insulin-like growth factor 1 receptor; Glut1, glucose transporter 1; Vegf, vascular endothelial growth factor; Pparγ, peroxisome proliferator-activated receptor gamma; Actb, beta-actin; Rn18s, 18S ribosomal RNA. (DOCX) [file pone.0328123.s001.docx]

| **Antibody Name** | **Target** | **Cat. Number** | **Dilution** | **Source** | **RIDD** |
| --- | --- | --- | --- | --- | --- |
| Anti-Carbonic Anhydrase 9/CA9 Antibody | CAIX | AF2344 | 1:200 | R&D Systems | AB_2244016 |
| Biotinylated anti-goat IgG |  | B7014 | 1:100 | Sigma-Aldrich | AB_258597 |

**Table S1**. A list of antibodies used for immunohistochemical analyses.
