# Supplementary material for: Prenatal cannabis smoke exposure alters placental development in a murine model of pregnancy
Source: PLoS One. 2026 Mar 16;21(3):e0328123. doi: 10.1371/journal.pone.0328123 (PMC12991273; doi:10.1371/journal.pone.0328123)
Supplement: S2 Table — (DOCX) [file pone.0328123.s002.docx]

**Table S2**. A list of mouse primer sequences used for RT-qPCR.

| **Gene** | **Sense Strand Primer Sequence (5’ 🡪 3’)** |
| --- | --- |
| Cyp1a1 | GCTTAGACTGTCCAGGATGC |
| Pl2 | CCAACGTGTGATTGTGGTGT |
| Tfap2c | CACCGTGACCCCGATTGT |
| Tpbpa | CCAGCACAGCTTTGGACATCA |
| Pcdh12 | CTCCTGTCCAGCAAATCTCC |
| Igf1r | GTTATCCACGACGATGAGTGC |
| Glut1 | TACACCCCAGAACCAATGGC |
| Vegf | TCATCAGCCAGGGAGTCTGT |
| Pparg | GCGGAAGAAGAGACCTGGG |
| Actb | AGCCATGTACGTAGCCATCCA |
| Rn18s | GTAACCCGTTGAACCCCATT |

^a^*Cyp1a1*, cytochrome P450 family 1 subfamily A polypeptide 1; *Pl2*, placental lactogen 2; *Tfap2c,* transcription factor AP-2 gamma; *Tpbpa*, trophoblast specific protein alpha; *Pcdh12*, procadherin 12; *Igf1r,* insulin-like growth factor 1 receptor; *Glut1*, glucose transporter 1; *Vegf*, vascular endothelial growth factor; *Pparγ*, peroxisome proliferator-activated receptor gamma; *Actb*, beta-actin; *Rn18s*, 18S ribosomal RNA.
