# Supplementary figures and images for: Prenatal cannabis smoke exposure alters placental development in a murine model of pregnancy
Source: PLoS One. 2026 Mar 16;21(3):e0328123. doi: 10.1371/journal.pone.0328123 (PMC12991273; doi:10.1371/journal.pone.0328123)

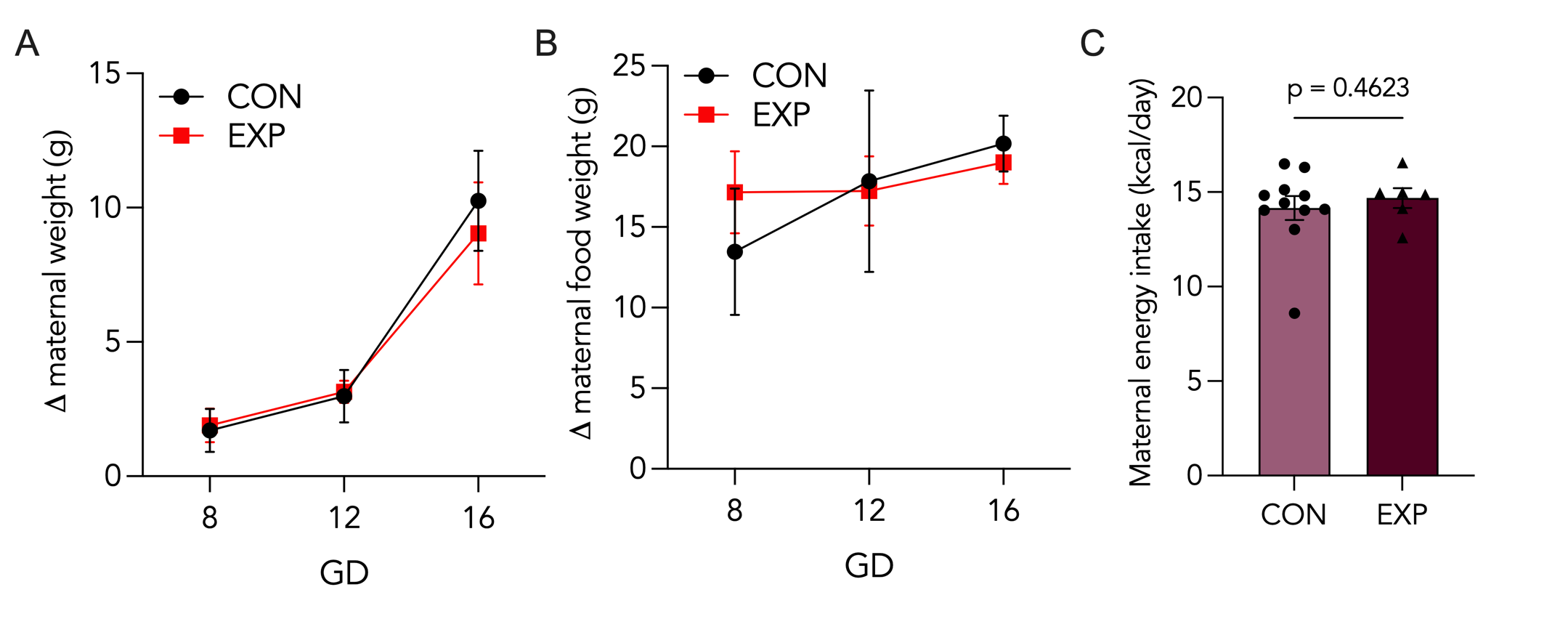

Supplement: S1 Fig — Control (CON) and cannabis smoke-exposed (EXP) dams weights and food weights were measured every four days. (A) Delta maternal weight gain and (B) food consumption (g) was quantified during pregnancy. (C) Estimated maternal daily energy intake based on the average food consumption over each four-day period per dam, according to the energy per gram of Teklad Global 19% Protein Extruded Diet (3.3kcal/g). Significant differences were determined by a Student’s t-test and are denoted by bolded p-values. (TIFF) [file pone.0328123.s003.tiff]

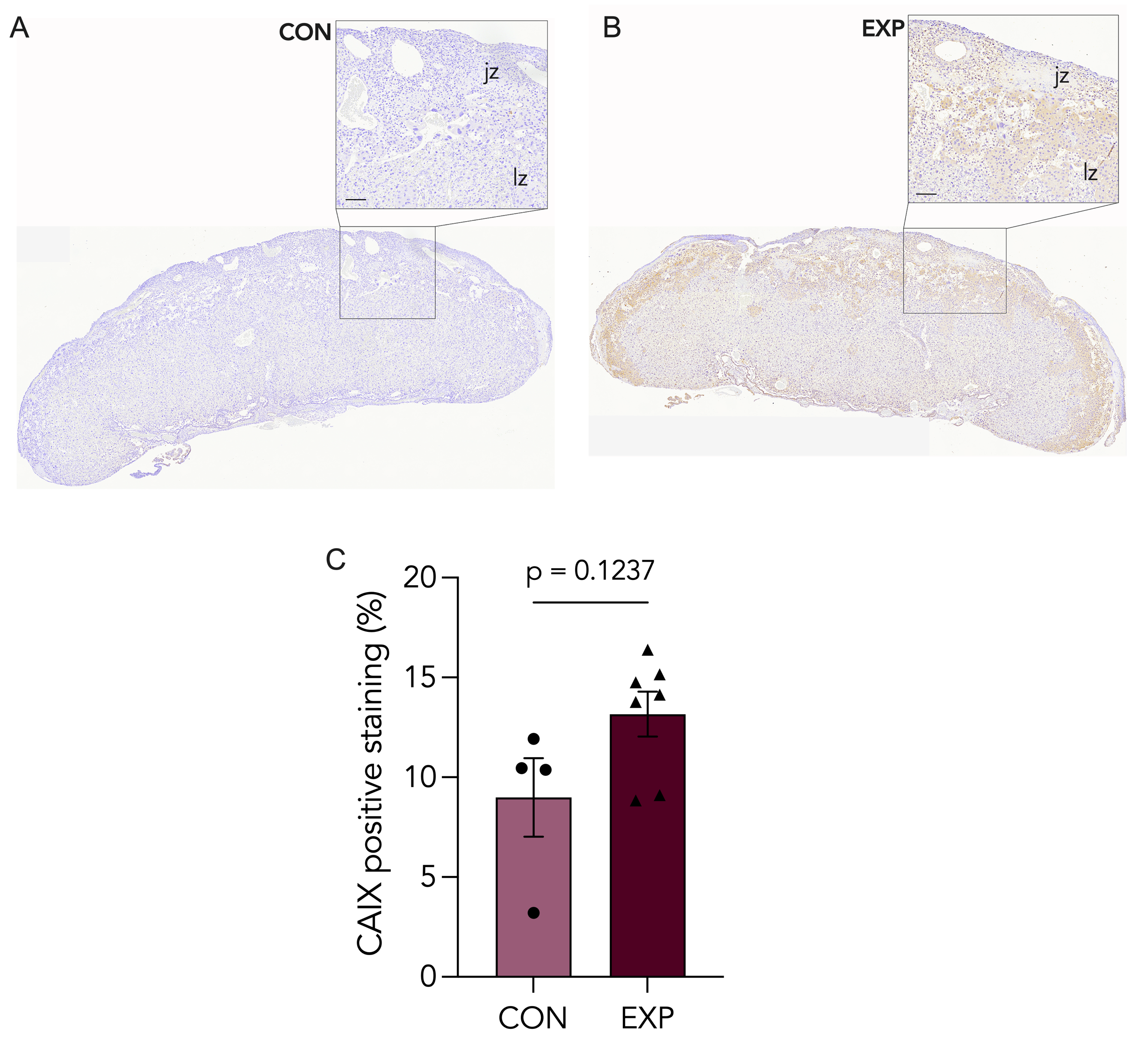

Supplement: S2 Fig — Representative images of whole E18.5 placentas from (A) control (CON; n = 4) and (B) cannabis smoke-exposed (EXP; n = 7) dams that were sectioned (5µm) and subsequently assessed for carbonic anhydrase (CAIX) expression with a polyclonal goat anti-carbonic anhydrase antibody (R&D Systems, AF2344) via immunohistochemistry. Magnification 400x, scale bars = 100µm. (C) Percent immunopositive area of CAIX staining from CON and EXP placentas at E18.5. Data are represented as a bar graph with each biological sample containing the average of CAIX quantification from two images per placenta. Statistical analyses included a Welch’s t-test. Significant differences are denoted by bolded p-values. Jz, junctional zone; Lz, labyrinth zone. (TIFF) [file pone.0328123.s004.tiff]

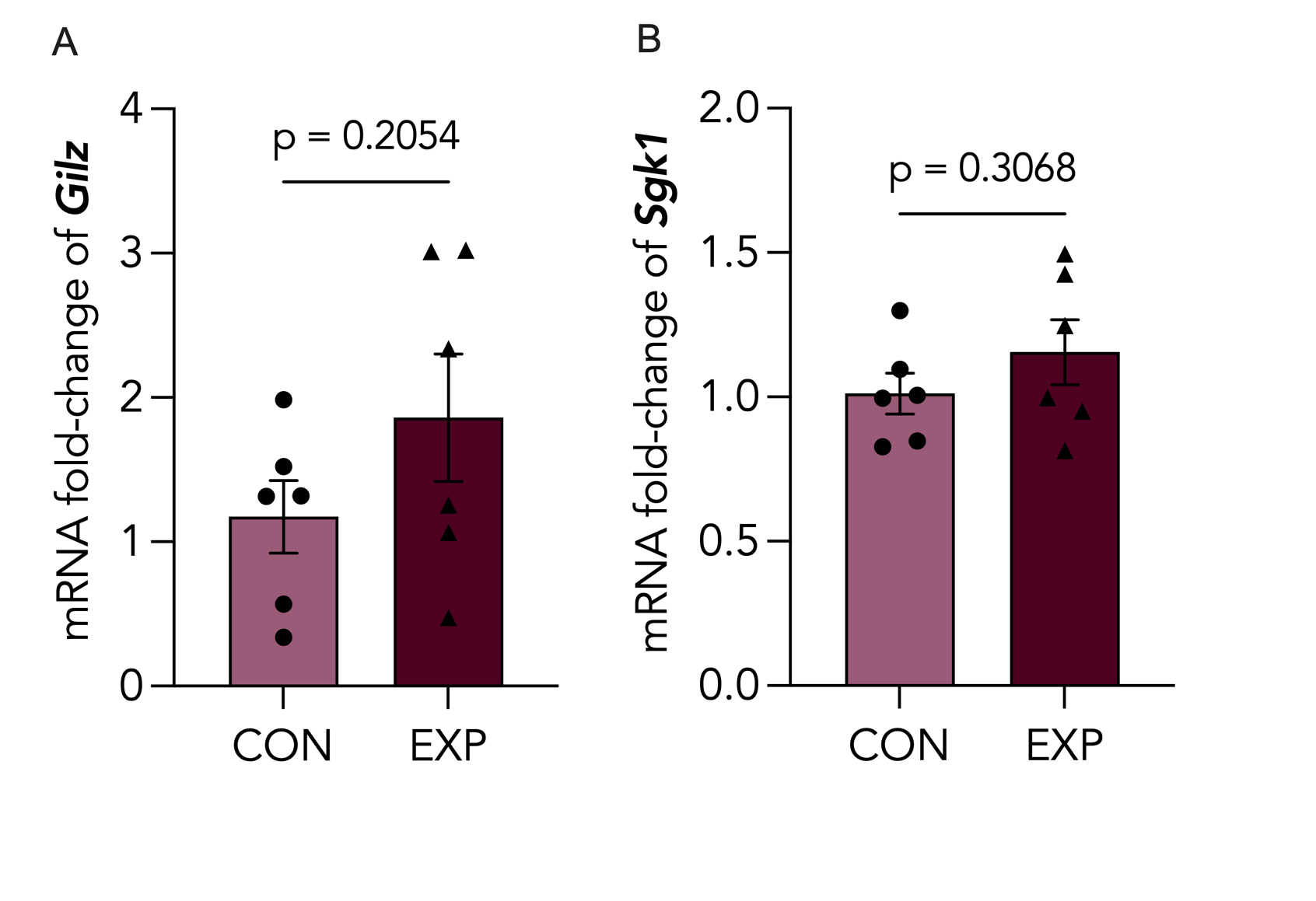

Supplement: S3 Fig — Control (CON) and cannabis-exposed (EXP) placentas were assessed for mRNA expression of (A) Gi1z and (B) Sgk1 via RT-qPCR and normalized to Actb and Rn18s. Data points represent the mean ± SEM of 6 biological replicates. Significant differences are denoted by bolded p-values. (TIFF) [file pone.0328123.s005.tiff]
